# Development of high‐resolution DNA barcodes for *Dioscorea* species discrimination and phylogenetic analysis

**DOI:** 10.1002/ece3.5605

**Published:** 2019-08-22

**Authors:** Wei Xia, Bo Zhang, Dan Xing, Ying Li, Wenqiang Wu, Yong Xiao, Jinhua Sun, Yajing Dou, Wenqi Tang, Jinlan Zhang, Xiaolong Huang, Yun Xu, Jun Xie, Jihua Wang, Dongyi Huang

**Affiliations:** ^1^ Hainan Key Laboratory for Sustainable Utilization of Tropical Bioresources College of Tropical Crops Hainan University Haikou China; ^2^ Coconut Research Institute CATAS Wenchang China; ^3^ Environment and Plant Protection Institute CATAS Haikou China; ^4^ Guangdong Key Laboratory for Crops Genetic Improvement Crops Research Institute Guangdong Academy of Agricultural Sciences Guangzhou China

**Keywords:** chloroplast genome, *Dioscorea*, DNA barcode, intergenic variation

## Abstract

The genus *Dioscorea* is widely distributed in tropical and subtropical regions, and is economically important in terms of food supply and pharmaceutical applications. However, DNA barcodes are relatively unsuccessful in discriminating between *Dioscorea* species, with the highest discrimination rate (23.26%) derived from *mat*K sequences. In this study, we compared genic and intergenic regions of three *Dioscorea* chloroplast genomes and found that the density of SNPs and indels in intergenic sites was about twice and seven times higher than that of SNPs and indels in the genic regions, respectively. A total of 52 primer pairs covering highly variable regions were designed and seven pairs of primers had 80%–100% PCR success rate. PCR amplicons of 73 *Dioscorea* individuals and assembled sequences of 47 *Dioscorea* SRAs were used for estimating intraspecific and interspecific divergence for the seven loci: The *rpo*B‐*trn*C locus had the highest interspecific divergence. Automatic barcoding gap discovery (ABGD), Poisson tree processes (PTP), and generalized mixed Yule coalescence (GMYC) analysis were applied for species delimitation based on the seven loci and successfully identified the majority of species, except for species in the *Enantiophyllum* section. Phylogenetic analysis of 51 *Dioscorea* individuals (28 species) showed that most individuals belonging to the same species tended to cluster in the same group. Our results suggest that the variable loci derived from comparative analysis of plastid genome sequences could be good DNA barcode candidates for taxonomic analysis and species delimitation.

## Graphical abstract

We compared genic and intergenic regions of three Dioscorea chloroplast genomes and seven pairs of primers had 80%–100% PCR success rate. Automatic barcoding gap discovery (ABGD), Poisson tree processes (PTP), and generalized mixed Yule coalescence (GMYC) analysis were applied for species delimitation based on the seven loci and successfully identified majority species based on more than 100 Dioscorea individuals from 30 species.

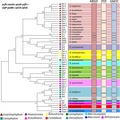

## 1 INTRODUCTION

The genus *Dioscorea* (family Dioscoreaceae) is comprised of approximately 630 species which are distributed across Southeast Asia, Africa, Central America, South America, and other tropical and subtropical regions. This genus is economically important for their tubers, which provide starch as a dietary staple as well as cortisone and other steroid hormones, such as dioscin (Aumsuwan et al., 2016; Cho et al., 2013; Jeon et al., 2006). However, *Dioscorea* species are hard to identify due to high morphological diversity, dioecy, small flowers, and morphological similarities between various species in this genus (Raman et al., 2014; Wilkin et al., 2005). Distinguishing *Dioscorea* species based on morphological traits is unreliable, while using DNA barcodes (*mat*K, *rbc*L, *psb*A‐*trn*H, *trn*L‐F) for *Dioscorea* species, identification has previously showed relatively low discrimination success, with the highest rate of 23.26% derived from use of the *mat*K sequences (Gao et al., 2008; Mukherjee & Bhat, 2013; Sun et al., 2012). Currently, chloroplast genome sequences of four species in the *Dioscorea* genus are available (Mariac et al., 2014; Wu et al., 2016; Zhou, Chen, Hua, & Wang, 2016), and thorough sequence comparison between these genomes could perhaps provide candidate regions for developing useful barcodes.

In the past decade, seven plastid DNA regions (*atp*F–*atp*H spacer, *mat*K gene, *rbc*L gene, *rpo*B gene, *rpo*C1 gene, *psb*K–*psb*I spacer, and *trn*H–*psb*A spacer) and 2‐locus combinations were frequently used to distinguish the land plants (Hollingsworth, Graham, & Little, 2011). To date, these DNA barcodes along with other barcodes such as *ycf*5, *psb*K‐I, *psb*M, *trn*D, and *rps*16 are still widely used for the identification of varieties and analysis of the provenances of varieties (Lee, Wang, Yen, & Chang, 2017; Techen, Parveen, Pan, & Khan, 2014). Moreover, new barcodes have been developed based on increasingly available sequence data and on deep mining for highly variable regions. Dong et al. (2015) were analyzed available plastid genomes and designed suitable primers for the most variable regions, and finally found that *ycf1*b generally performed better than any of the *mat*K, *rbc*L, and *trn*H‐*psb*A barcodes. Among 18 *Oryza* chloroplast genomes, five variable regions (*rps16*‐*trnQ*, *trnTEYD*, *psbE*‐*petL*, *rpoC2*, and *rbcL*‐*accD*) were analyzed for species discrimination (Song et al., 2017). However, systematic comparisons for plastid genome sequences have not been conducted between *Dioscorea* species and would provide useful information for identifying better‐performing DNA barcodes for *Dioscorea* species.

In this study, we downloaded plastid genome sequences for three *Dioscorea* species—*D. rotundata*, *D. elephantipes*, and *D. zingiberensis*—and made a comprehensive comparison of the genic and intergenic regions to characterize conserved regions and variable regions. Top variable regions were selected and covered by 52 pairs of primers, and we tested primer universality in 10 *Dioscorea* species. Moreover, 47 sequence read archives (SRAs) for 18 *Dioscorea* species were also downloaded and assembled for the corresponding plastid sequences for our selected variable regions. This study aimed to develop efficient DNA barcodes for *Dioscorea* species discrimination and to provide useful information for further DNA barcode development.

## 2 MATERIALS AND METHODS

### 2.1 Plant materials

**Figure 1:**
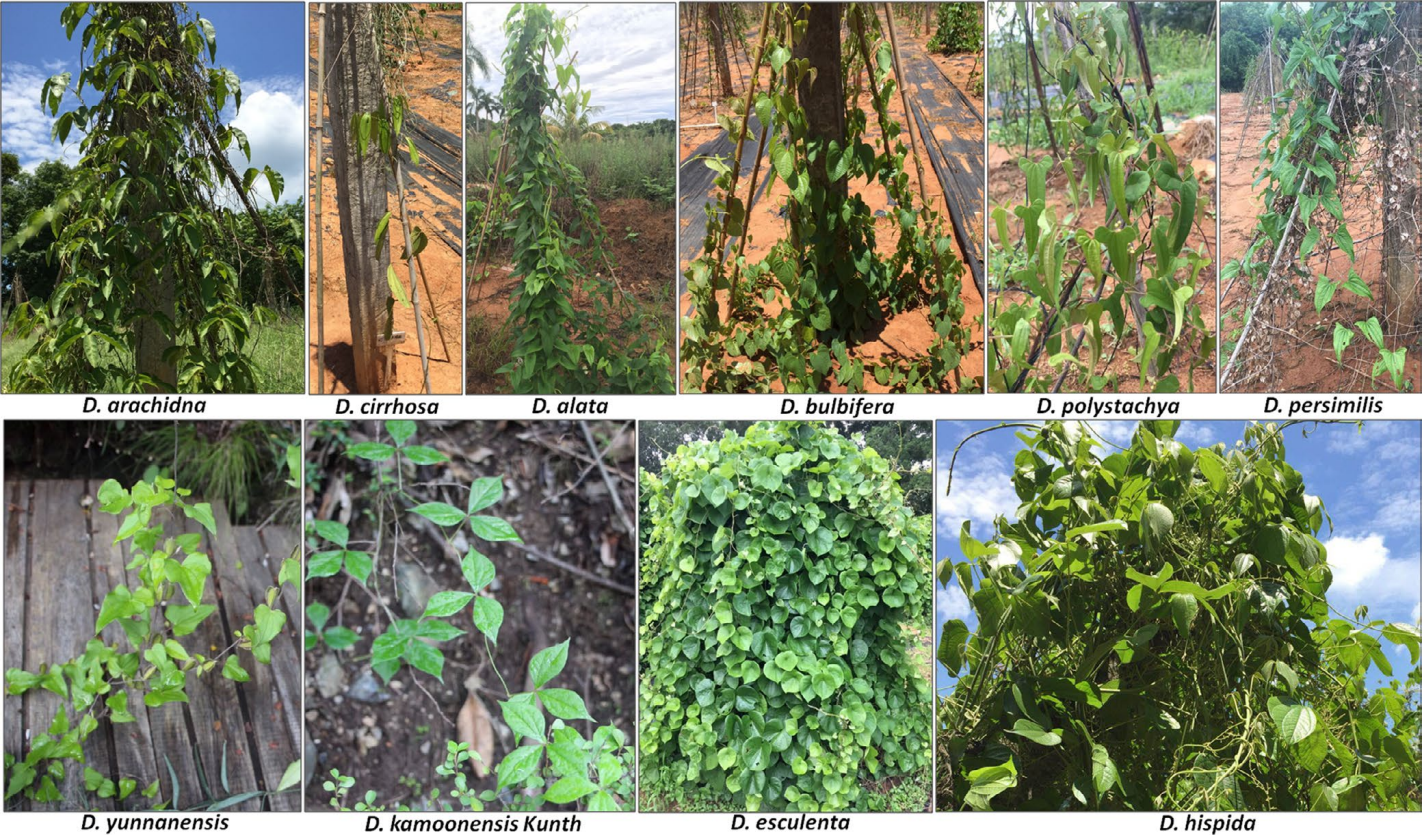
Representative plant individuals of the 10 *Dioscorea* species used in this study

Plant samples were collected from different provinces in China and were kept in the *Dioscorea* germplasm nursery of Danzhou, Hainan, China. A total of 74 individuals belonging to 10 species and representing a high number of economically useful plants were used for this study. The individual images of the 10 *Dioscorea* species (*D. alata*, *D. polystachya*, *D. esculenta*, *D. persimilis*, *D. bulbifera*, *D. cirrhosa*, *D. hispida*, *D. arachidna*, *D. kamoonensis* Kunth, and *D. yunnanensis*) are shown in Figure 1. Detailed information for these analyzed *Dioscorea* species is listed in Table S1. Fresh leaves were used to extract DNA.

### 2.2 Sequence analysis for plastid genome of *Dioscorea* species and primer design

The chloroplast genome sequences for *D. rotundata*, *D. elephantipes*, and *D. zingiberensis* were downloaded from the National Center for Biotechnology Information (NCBI; https://www.ncbi.nlm.nih.gov/). We used BLAST to align the genic and intergenic regions of the three plastid sequences. Divergent hot regions were identified, and a set of primers were designed to cover these plastid regions (Table S2). The primer design was using the software—Primer Premier 5.

A total of 47 SRAs for 18 *Dioscrea* species (*D. baya*, *D. burkilliana*, *D. cayennensis*, *D. dumetorum*, *D. hirtiflora*, *D. minutiflora*, *D. preussii*, *D. quartiniana*, *D. sagittifolia*, *D. sansibarensis*, *D. schimperiana*, *D. smilacifolia*, *D. togoensis*, *D. villosa*, *D. bulbifera*, *D. rotundata*, *D. abyssinica*, and *D. praehensilis*) locating in Liberia, Cameroon, Republic of the Congo, Gabon, Ethiopia, Benin, Senegal, Guinea, Ghana, Malawi, Cote d'Ivoire, Togo, USA, and Benin were downloaded from the NCBI, and detailed information for the datasets and the species were listed in Table S3. Based on the plastid genome sequences for the above three *Dioscorea* species, the mapping software bowtie2 (Langmead & Salzberg, 2012) was used to identify plastid‐related sequences. The mapped reads were assembled with CAP3 (Huang & Madan, 1999). We applied the NCBI Primer‐BLAST to test the efficiency of primers.

### 2.3 DNA extraction, amplification, and sequencing

DNA extraction was following a cetyl trimethylammonium bromide (CTAB) protocol modified from Paterson, Brubaker, and Wendel (1993). One individual for each of the 10 *Dioscorea* species we sampled was used to select primers and test the amplification efficiency. The polymerase chain reaction (PCR) mixture contained 4 μl diluted DNA (50 ng/μl), 10 μl of 2 × Mix (Yugong Biolab), and 1 μl of each forward and reverse primer (10 μM) in a final volume of 20 μl. PCR amplification was carried out under following conditions: 5 min at 94°C, and 32 cycles of 30 s at 94°C, 30 s at 55°C, 1 min at 72°C, and a final step of 7 min at 72°C. PCR products were examined electrophoretically on 2% agarose gels. Purification and sequencing were done by Guangzhou Tianyi Huiyuan Biological Technology Company using the amplification primers. The nucleotide sequence data were deposited in the European nucleotide Archive database (Table S1).

### 2.4 Sequence alignment and data analysis for DNA barcode

All sequences were aligned and adjusted manually by MEGA 7.0 (Kumar, Stecher, & Tamura, 2016), and all variable sites for these sequences obtained by sequencing in this study were rechecked on the original trace files for final confirmation. Both concatenated dataset and single locus sequences were applied for phylogenetic tree construction. The phylogenetic trees were constructed using maximum likelihood (ML), and node support was assessed by a bootstrap test (1,000 pseudoreplicates of run with K2P distance as a model of substitution). All genetic distances were calculated in MEGA 7.0. Average intraspecific distance and interspecific distance were calculated to determine interspecific and intraspecific divergence, respectively. Wilcoxon signed‐rank tests for the interspecific divergences among the selected barcode loci were performed by SPSS (IBM Corp, 2017).

We used three independent species delimitation approaches, automatic barcoding gap discovery (ABGD, Puillandre, Lambert, Brouillet, & Achaz, 2012), Poisson tree processes (PTP, Zhang, Kapli, Pavlidis, & Stamatakis, 2013), and generalized mixed Yule coalescence (GMYC, Suchard et al., 2018), to determine putative molecular species in our dataset and evaluate the performance of the selected barcode loci. Matrices of pairwise genetic distances using the p‐distance, the Kimura 2‐parameter (K2P), and the Jukes‐Cantor (JC69) models were computed by MEGA 7.0 and used as input files on the ABGD webpage (http://wwwabi.snv.jussieu.fr/public/abgd/abgdweb.html). We set parameters as follows: *P*_min_ = 0.001, *P*_max_ = 0.01, Steps = 50, *X* = 1.0, and Nb bins = 20. We performed PTP analyses on the bPTP web server (http://species.h-its.org/ptp/) with the RAxML topology (Kozlov, Darriba, Flouri, Morel, & Stamatakis, 2019) and used the 50% majority‐rule consensus topology resulting from the BI analysis as output files. We ran PTP analyses for 400,000 MCMC generations, set the thinning value = 100 and burn‐in = 0.25. We visually confirmed the convergence of the MCMC chain as recommended by Zhang et al. (2013). We used ultrametric trees generated with BEAST 1.10.4 for GMYC analyses (Suchard et al., 2018). The ultrametric trees were constructed as follows: Coalescent tree prior and the heterogeneity of the mutation rate across lineages were set under an uncorrelated lognormal relaxed clock. The analysis was run for 100 million generations with a sampling frequency of 10,000. After checking adequate mixing and convergence of all runs with Tracer 1.7.1 (Rambaut, Drummond, Xie, Baele, & Suchard, 2018), the first 25% trees were discarded as burn‐in. The maximum clade credibility tree was computed using TreeAnnotator 1.10.4 (Suchard et al., 2018). The resulting ultrametric tree was imported into R 3.6.0 (R Core Team, 2018), and GMYC analyses were run using the Splits (Ezard, Fujisawa, & Barraclough, 2009) and Ape (Paradis, Claude, & Strimmer, 2004) libraries.

## 3 RESULTS

### 3.1 Chloroplast genome sequence divergence in three *Dioscorea* species

To identify suitable sequences for species discrimination in *Dioscorea* species, chloroplast genome sequences for *D. elephantipes*, *D. rotundata*, and *D. zingiberensis* were downloaded from the NCBI website and analyzed. The three chloroplast genomes ranged from 152,609 bp (*D. elephantipes*) to 155,406 bp (*D. rotundata*), consisting of a pair of inverted repeats (25,476–25,509 bp) separated by the long single copy section (80,777–85,601 bp) and short single copy section (18,814–19,038 bp) regions (Table S4). All three chloroplast genomes had the same gene number (140), including 94 protein‐coding genes, 38 tRNA genes, and eight rRNA genes.

Alignments for 140 genic sequences from the three chloroplast genomes showed that the three *Dioscorea* species have 109,121–109,989 aligned genic sequence and shared high sequence similarities (87%–100%) (Table 1). Moreover, genic sequences between *D. elephantipes* and *D. rotundata* showed higher similarity (96%–100%), with fewer nucleotide polymorphisms (SNPs) and indels than the other two interspecies comparisons. More than 20/1,000 nucleotide variations were detected between *D. zingiberensis* and either of the other two species, while <10/1,000 nucleotide variations were detected between *D. elephantipes* and *D. rotundata* (Table 1). The total length of indel sites was 10 times less than that of the SNPs, with 94–183 bp of indels identified between the three species. The intergenic sequence alignments between the three *Dioscorea* species indicated lower sequence similarity for the most variable regions (76%–79%) than the genic sequence alignments (87%–96%). The densities of SNPs and indels in intergenic sites were about twice and seven times higher, respectively, than the densities of SNPs and indels in the genic regions. About 49–57 per 1,000 nucleotides variations were detected between *D. zingiberensis* and either of the other two species, while <26 per 1,000 nucleotide variations were detected between *D. elephantipes* and *D. rotundata*.

**Table 1:** Genic and intergenic variation between *Dioscorea elephantipes* (Del), *D. rotundata* (Dro), and *D. zingiberensis* (Dzi) based on nucleotide BLAST

Compared species		Genic region					Intergenic region					Length ratio (Intergenic/genic)	
		Aligned length (bp)	Similarity	Variable sites			Aligned length (bp)	Similarity	Variable sites			SNP	Indel
				SNP (bp)	Indel (bp)	Total %			SNP (bp)	Indel (bp)	Total %		
Del	Dro	109,121	96%−100%	890	94	0.93	44,717	79%−100%	901	287	2.66	2.5	7.5
Dzi	Dro	109,295	87%−100%	2,387	175	2.41	40,055	77%−100%	1,802	504	5.76	2.1	7.9
Del	Dzi	109,989	87%−100%	2,049	183	2.15	44,614	76%−100%	1,693	495	4.90	2.0	6.7

Top variable genic and intergenic regions were selected mainly based on the number of variable sites and listed in Table 2 with seven widely used DNA barcode markers. The seven DNA barcodes including four genic regions (*mat*K, *rbc*L, *rpo*B, and *rpo*C1 genes) and three intergenic regions (*atp*F–*atp*H spacer, *psb*K–*psb*I spacer, and *trn*H–*psb*A spacer) showed a lower frequency of variation between the three *Dioscorea* species. The number of variable sites for *rbc*L (23), *mat*K (30), and *rpo*B (30) genes was less or close to those of the seven other variant genic regions (30–118), and the frequency of variants in the *rpo*B (0.93%) and *rpo*C1 (1.32%) genes was close to the average variation frequency (0.93%) between *D. elephantipes* and *D. rotundata* (Table 2). The frequency of the variable sites for *rbc*L, *rpo*B, and *rpo*C1 was lower than that of most other variable genic regions between *D. zingiberensis* and the two other species. However, the *mat*K gene showed similar variant frequency when compared to other variable genic regions. The *atp*F–*atp*H spacer, *psb*K–*psb*I spacer, and *trn*H–*psb*A showed less variable sites and lower variable frequency than most other variable intergenic regions (Table 2).

**Table 2:** Candidate DNA barcode regions with high variations between *Dioscorea elephantipes* (Del), *D. rotundata* (Dro), and *D. zingiberensis* (Dzi)

Regions with high variation	Length	Variable sites					
		Del‐Dro		Dzi‐Dro		Del‐Dzi	
		Numbers	%	Numbers	%	Numbers	%
Genic							
***rbc*L**	1,434	23	1.60	37	2.6	25	1.74
*ndh*F	2,250	29	1.29	116	5.2	102	4.60
***mat*K**	1,560	30	1.92	76	4.9	69	4.43
*atp*F	1,576	30	1.94	91	5.8	76	4.82
***rpo*B**	3,213	30	0.93	76	2.4	67	2.09
*rpl*16	1,463	31	2.15	89	6.1	82	5.60
***rpo*C1**	2,089	37	1.32	87	3.1	86	3.06
*clp*P	2,025	39	1.96	92	4.6	86	4.25
*ndh*A	2,190	40	1.84	101	4.6	78	3.56
*rpo*C2	4,153	49	1.18	126	3.0	109	2.63
*ycf*1	5,629	118	2.11	316	5.6	288	5.12
Intergenic							
***trn*H*‐psb*A**	295	3	1.02	13	4.6	15	5.38
***psb*K*‐psb*I**	417	9	2.19	30	7.2	31	7.43
***atp*F*‐atp*H**	261	9	3.45	7	6.1	10	5.95
*psa*A*‐ycf*3	638	19	3.11	38	6.0	33	5.33
*clp*P*‐psb*B	508	21	4.27	24	4.7	27	5.33
*trn*E*‐trn*T	838	23	2.96	54	6.4	50	6.01
*psb*E*‐pet*L	685	28	4.09	52	8.3	43	6.95
*trn*T*‐psb*D	1,114	34	3.85	48	7.4	77	6.91
*trn*L*‐rpl*32	915	35	4.00	116	12.7	113	12.39
*trn*T*‐trn*L	927	39	4.68	64	6.9	57	6.22
*trn*C*‐pet*N	1,056	40	4.01	71	6.8	66	6.25
*ycf*4*‐cem*A	695	41	6.56	34	6.8	62	8.92
*rpo*B*‐trn*C	1,378	57	4.47	116	8.4	81	5.92
*ndh*C*‐trn*V	1,019	62	6.14	90	8.8	56	5.58
*trn*K*‐trn*Q	1,653	68	4.11	77	7.4	77	7.35
*trn*S*‐trn*G	1,091	76	7.20	147	13.8	125	11.46
*rpl*32*‐ndh*F	790	96	12.15	16	6.1	31	6.97

Note

The classic DNA barcodes are in bold font.

### 3.2 Seven highly variable regions for candidate DNA barcodes

To develop DNA barcode for *Dioscorea* species discrimination, 52 primer pairs covering highly variable regions were designed (Table S2), including the top variable regions in Table 2. One individual from each of the 10 *Dioscorea* species (*D. alata*, *D. polystachya*, *D. esculenta*, *D. persimilis*, *D. bulbifera*, *D. cirrhosa*, *D. hispida*, *D. arachidna*, *D. kamoonensis* Kunth, and *D. yunnanensis*), which belong to five *Dioscorea* sections—Enantiophyllum Uline (4), Combilium Prain et Burkill (1), Opsophyton Mine (1), Lasiophyton Uline (3), and Shannicorea Prain et Burkill (1) (Table S1), was used for selecting primers with high universality. The PCR results showed that 11/52 pairs of primers had positive amplification products. Moreover, seven pairs of primers covering one genic sequence—*atp*F, four intergenic sequences—*rpo*B*‐trn*C, *ycf*4*‐cem*A, *clp*P*‐psb*B, and *rpl*14*‐rpl*16, and two containing both genic and intergenic sequences—*trn*D*‐trn*T and *psa*A*‐ycf*3 had 80%–100% PCR success rate, while the other four primer pairs primer successfully amplified only in one to two species.

We also tested the primers for the assembled sequences from 47 individuals, which belong to 18 *Dioscorea* species: *D. baya*, *D. burkilliana*, *D. cayennensis*, *D. dumetorum*, *D. hirtiflora*, *D. minutiflora*, *D. preussii*, *D. quartiniana*, *D. sagittifolia*, *D. sansibarensis*, *D. schimperiana*, *D. smilacifolia*, *D. togoensis*, *D. villosa*, *D. bulbifera*, *D. rotundata*, *D. abyssinica*, and *D. praehensilis*. These species belong to seven *Dioscorea* sections: Enantiophyllum Uline (10), Lasiophyton Uline (1), Asterotricha (2), Macrocarpaea (1), Botryosicyos (1), Macroura (1), and Opsophyton Mine (1) (Table S3). The ePCR results showed that the seven pairs of primers have ePCR success rates as 77%–100%, which were similar to the PCR results for the 10 *Dioscorea species* (Table 3). The primers for *psa*A*‐ycf*3 and *ycf*4*‐cem*A have the top ePCR success rate, followed by *trn*D‐*trn*T and *clp*P‐*psb*B with ePCR success rate as 94% (17/18). Combined with the above PCR results, *psa*A*‐ycf*3 and *ycf*4*‐cem*A were still the top primers with 100% ePCR success rate (Table 3).

**Table 3:** Variability of the seven new markers and the DNA barcodes in *Dioscorea* species

Markers	Length (bp)		Conserved sites (bp)	Variable sites (bp)			Amplification efficiency^a^	ePCR efficiency^b^
	Sequence	Aligned		Indels	SNPs	Total		
*atp*F	478–636	678	408	223	47	270	9/10	14/18
*rpo*B‐*trn*C	567–664	710	459	112	139	251	10/10	15/18
*trn*D*‐trn*T	860–873	895	778	60	57	117	8/10	17/18
*psa*A*‐ycf*3	774–927	968	664	218	86	304	10/10	18/18
*ycf*4*‐cem*A	293–916	946	260	660	26	686	10/10	18/18
*clp*P*‐psb*B	843–880	906	753	96	57	153	10/10	17/18
*rpl*14*‐rpl*16	858–889	912	677	93	142	235	10/10	16/18

a PCR amplification conducted in 10 *Dioscorea* species (*D. alata*, *D. polystachya*, *D. esculenta*, *D. persimilis*, *D. bulbifera*, *D. cirrhosa*, *D. hispida*, *D. arachidna*, *D*. *kamoonensis* Kunth, and *D. yunnanensis*).

b Primer‐BLAST analysis conducted in assembled sequences for 18 *Dioscorea* species (*D. baya*, *D. burkilliana*, *D. cayennensis*, *D. dumetorum*, *D. hirtiflora*, *D. minutiflora*, *D. preussii*, *D. quartiniana*, *D. sagittifolia*, *D. sansibarensis*, *D. schimperiana*, *D. smilacifolia*, *D. togoensis*, *D. villosa*, *D. bulbifera*, *D. rotundata*, *D. abyssinica*, and *D. praehensilis*).

We aligned sequences from PCR amplification and the assembled sequences for the seven regions with high PCR success rate. The PCR amplicons ranged from 293 to 927 bp in size after trimming flanking sequences with low quality. A total of 29 *Dioscorea* species belonging to eleven sections were analyzed, including plastid sequences from *D. elephantipes* and *D. zingiberensis*. Multiple sequence alignments showed that these sequences have ample indels covering 60–660 bp and 26–142 SNPs for these amplicons between the *Dioscorea* species (Table 3 and Figure S1). The indels identified in these amplicons were consistent within species. The sequences for *atp*F, *psa*A‐*ycf*3, and *ycf*4*‐cem*A contained an indel site more than 100 bp and distinct sequence divergences between *Dioscorea* species were detected (Figure S1). *D. yunnanensis* (Dy1, Dy2 and Dy3) had the longest deletion: 174 bp in this indel region of *atp*F and eleven distinct types of sequences existed, while eight types of sequences were observed in the indel region of *psa*A*‐ycf*3. However, the large indel regions of *atp*F and *psa*A*‐ycf*3 for *D. hispida* and *D. arachidna* were indistinguishable (Figure S1). A 645 bp deletion was detected for the *ycf4‐cemA* region in *D. cirrhosa*, followed with a 182 bp deletion in *D. arachidna*. Eleven distinct types of sequences existed in this indel region of *ycf*4*‐cem*A, but *D. alata* and *D. hispida* were indistinguishable. Besides, the *rpo*B‐*trn*C (139) and *rpl*14‐*rpl*16 (142) contained more SNPs than the other five regions.

Analysis of intraspecific and interspecific distances showed that *rpl*14‐*rpl*16 locus had the highest intraspecific distance (maximum = 0.044; mean = 0.004), and the remaining loci presented an average intraspecific distances as 0.001–0.002 (Table 4). The intergenic region (*rpo*B‐*trn*C) showed the greatest mean interspecific divergence (0.037), followed by *atp*F (0.027) and *rpl*14‐*rpl*16 (0.024). The noncoding region (*trn*D‐*trn*T) had the smallest average interspecific divergence (0.013). Wilcoxon signed‐rank tests demonstrated that the *rpo*B‐*trn*C had significantly higher interspecific divergence than that of other species, and the locus with the second highest interspecific divergence is *atp*F, while *rpl*14‐*rpl*16, *ycf*4‐*cem*A, and *psa*A‐*ycf*3 had similar interspecific divergences (Table 5).

**Table 4:** The pairwise intraspecific and interspecific distances for seven variable loci in *Dioscorea* species and species delimitation analysis through automatic barcoding gap discovery (ABGD), Poisson tree processes (PTP), and generalized mixed Yule coalescence (GMYC) analysis

Loci	Intraspecific distances			Interspecific distances			No. of species^a^	ABGD analysis^b^		PTP analysis^c^	GMYC analysis^d^
	Minimum	Maximum	Mean	Minimum	Maximum	Mean					
atpF	0.000	0.002	0.001	0.000	0.093	0.027	25	22 (0.1%)	13 (0.3%)	11	16 (.05)
rpoB‐trnC	0.000	0.013	0.002	0.002	0.104	0.037	21	27 (0.1%)	17 (0.3%)	36	27 (.001)
trnD‐trnT	0.000	0.014	0.001	0.000	0.025	0.013	25	22 (0.1%)	20 (0.2%)	20	22 (9.2E‐06)
psaA‐ycf3	0.000	0.012	0.001	0.000	0.063	0.018	29	39 (0.1%)	20 (0.3%)	36	45 (.01)
ycf4‐cemA	0.000	0.012	0.001	0.000	0.080	0.021	30	31 (0.1%)	15 (0.6%)	35	29 (0.001)
clpP‐psbB	0.000	0.005	0.001	0.000	0.041	0.013	28	26 (0.1%)	2 (2.5%)	20	26 (.01)
rpl14‐rpl16	0.000	0.044	0.004	0.000	0.110	0.024	28	32 (0.1%)	8 (1.7%)	24	26 (.002)

a Number of *Dioscorea* species with full‐length corresponding sequences used in the analysis.

b The values outside the brackets represent the numbers of estimated species; barcode gap values are inside the brackets. Two barcode gap thresholds are displayed.

c Number of estimated species with support values higher than 0.5.

d The values outside the brackets represent the number of estimated species, and the *p* value is inside the brackets.

**Table 5:** Wilcoxon signed‐rank test of interspecific divergence between the seven loci

*W*+	*W*−	Relative ranks, *n*, *p* value^a^	Result
*atp*F	*clp*P‐*psb*B	*W*+ = 37,033, *W*− = 2,588, 300, *p* ≤ .000	*atp*F > *clp*P‐*psb*B
*atp*F	*rpl*14‐*rpl*16	*W*+ = 25,879, *W*− = 16,899, 300, *p* ≤ .002	*atp*F > *rpl*14‐*rpl*16
*atp*F	*ycf*4‐*cem*A	*W*+ = 21,888, *W*− = 11,265, 276, *p* ≤ .000	*atp*F > *ycf*4‐*cem*A
*atp*F	*rpo*B‐*trn*C	*W*+ = 4,612.5, *W*− = 9,922.5, 171, *p* ≤ .000	*atp*F < *rpo*B‐*trn*C
*atp*F	*trn*D‐*trn*T	*W*+ = 18,908.5, *W*− = 4,527.5, 231, *p* ≤ .000	*atp*F > *trn*D‐*trn*T
*atp*F	*psa*A‐*ycf*3	*W*+ = 29,502.5, *W*− = 10,683.5, 300, *p* ≤ .000	*atp*F > *psa*A‐*ycf*3
*rpl*14‐*rpl*16	*clp*P‐*psb*B	*W*+ = 47,683.5, *W*− = 13,741.5, 378, *p* ≤ .000	*rpl*14‐*rpl*16 > *clp*P‐*psb*B
*rpl*14‐*rpl*16	*ycf*4‐*cem*A	*W*+ = 32,662, *W*− = 32,662, 378, *p* = .31	*rpl*14‐*rpl*16 = *ycf*4‐*cem*A
*rpl*14‐*rpl*16	*rpo*B‐*trn*C	*W*+ = 5,575, *W*− = 12,570, 190, *p* ≤ .000	*rpl*14‐*rpl*16 < *rpo*B‐*trn*C
*rpl*14‐*rpl*16	*trn*D‐*trn*T	*W*+ = 26,095, *W*− = 18,158, 300, *p* ≤ .000	*rpl*14‐*rpl*16 > *trn*D‐*trn*T
*rpl*14‐*rpl*16	*psa*A‐*ycf*3	*W*+ = 24,304, *W*− = 27,699, 325, *p* = .31	*rpl*14‐*rpl*16 = *psa*A‐*ycf*3
*rpo*B‐*trn*C	*clp*P‐*psb*B	*W*+ = 17,966, *W*− = 179, 190, *p* ≤ .000	*rpo*B‐*trn*C > *clp*P‐*psb*B
*rpo*B‐*trn*C	*ycf*4‐*cem*A	*W*+ = 13,736, *W*− = 4,409, 190, *p* ≤ .000	*rpo*B‐*trn*C > ycf4‐cemA
*rpo*B‐*trn*C	*trn*D‐*trn*T	*W*+ = 8,706, *W*− = 610, 136, *p* ≤ .000	*rpo*B‐*trn*C > *trn*D‐*trn*T
*rpo*B‐*trn*C	*psa*A‐*ycf*3	*W*+ = 12,783, *W*− = 1,923, 171, *p* ≤ .000	*rpo*B‐*trn*C > *psa*A‐*ycf*3
*ycf*4‐*cem*A	*clp*P‐*psb*B	*W*+ = 56,159, *W*− = 12,847, 378, *p* ≤ .000	*ycf*4‐*cem*A > *clp*P‐*psb*B
*ycf*4‐*cem*A	*trn*D‐*trn*T	*W*+ = 25,155, *W*− = 17,916, 300, *p* = .013	*ycf*4‐*cem*A > *trn*D‐*trn*T
*ycf*4‐*cem*A	psaA‐ycf3	*W*+ = 25,050, *W*− = 25,990, 300, *p* = .776	*ycf*4‐*cem*A = *psa*A‐*ycf*3
*clp*P‐*psb*B	*trn*D‐*trn*T	*W*+ = 9,684, *W*− = 33,387, 300, *p* ≤ .000	*clp*P‐*psb*B < *trn*D‐*trn*T
*clp*P‐*psb*B	*psa*A‐*ycf*3	*W*+ = 3,243, *W*− = 47,478, 325, *p* ≤ .000	*clp*P‐*psb*B < *psa*A‐*ycf*3
*trn*D‐*trn*T	*psa*A‐*ycf*3	*W*+ = 11,536, *W*− = 244,779, 276, *p* ≤ .000	trnD‐trnT < *psa*A‐*ycf*3

a The symbols “*W*+” and “*W*−” represent the sum of all of the positive values and the sum of all of the negative values in the signed‐rank column, respectively.

### 3.3 Applicability for species discrimination

A total of 73 individuals belonging to 10 *Dioscorea* species (Table S1), a set of 18 *Dioscorea* species with available SRAs (Table S3), and the three *Dioscorea* species with complete plastid genomes were used for estimation of species discrimination efficiency of the above loci. A total of 11 sections of *Dioscorea* species were included in this analysis, including *Enantiophyllum*, *Shannicorea*, *Asterotricha*, *Lasiophyton*, *Macrocarpaea*, *Lasiophyton*, *Testudinana*, *Combilium*, *Stenophora*, *Macroura*, and *Botryosicyos* (Tables S1 and S3).

**Figure 2:**
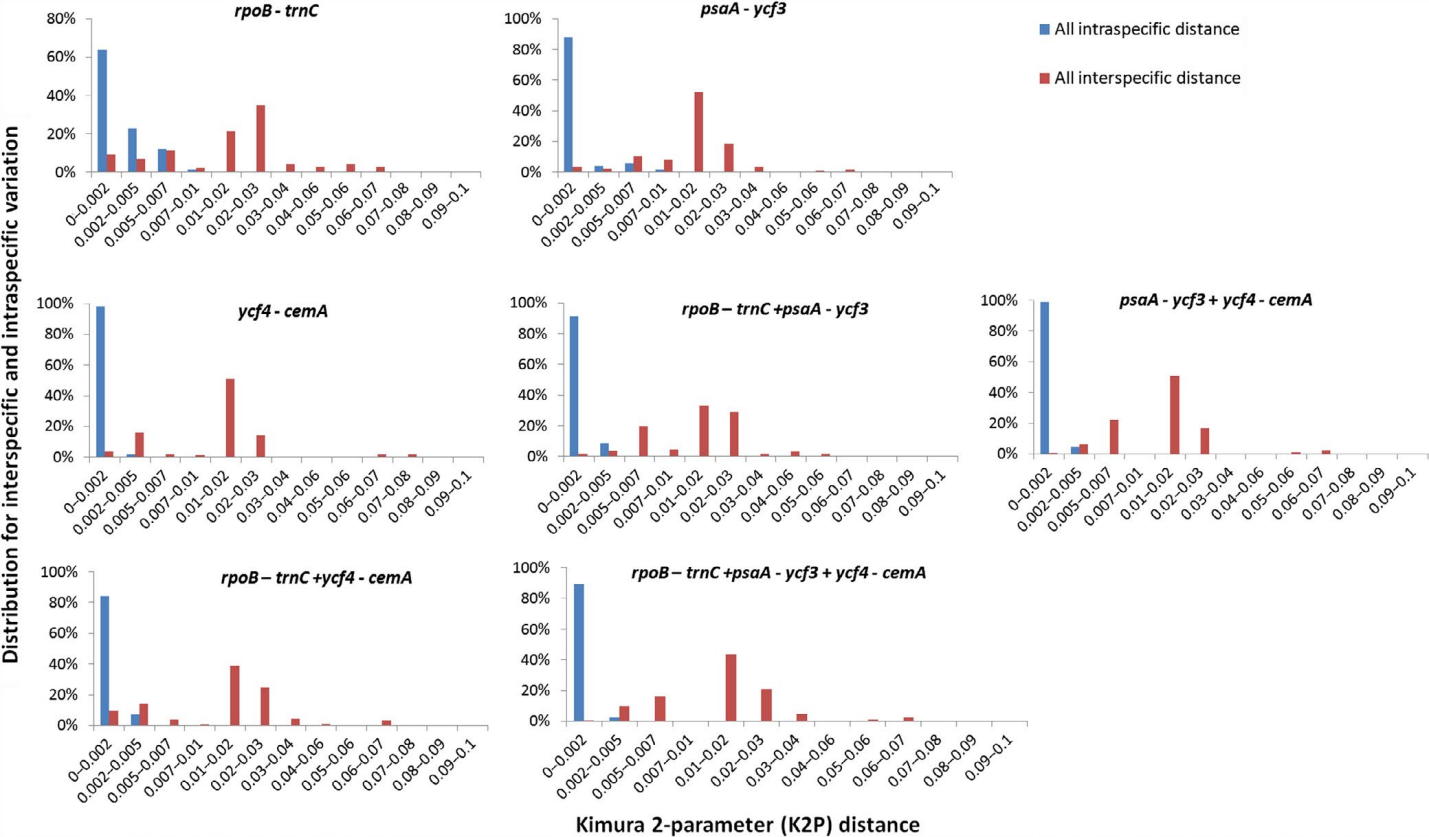
Relative distribution of interspecific divergence between congenic species and intraspecific variation

Measuring the intraspecific variation and interspecific divergence showed intraspecific variations for the seven loci were much lower than interspecific divergences (Figure 2). The majority of intraspecific variation for *atp*F (92%), *psa*A‐*ycf*3 (92%), and *ycf*4‐*cem*A (98%) were in sections 0–0.002, while *clp*P‐*psb*B (77%), *trn*D‐*trn*T (76%), *rpl*14‐*rpl*16 (64%), and *rpo*B‐*trn*C (63%) had relatively smaller proportions of intraspecific variation in sections 0–0.002. Interspecific variations were mainly >0.01, including for *rpo*B‐*trn*C (93%), *atp*F (79%), *ycf*4‐*cem*A (72%), *psa*A‐*ycf*3 (70%), *trn*D‐*trn*T (68%), *rpl*14‐*rpl*16 (62%), and *clp*P‐*psb*B (56%). Based on ABGD analysis, *psaA*‐*ycf3* had the highest estimated number of species discriminated (39) with a low threshold value (0.1%), followed by *rpl*14‐*rpl*16 (31) and *ycf*4‐*cem*A (32) (Table 4). When the barcode gap was set as 0.3%–0.6%, both *psa*A‐*ycf*3 and *trn*D‐*trn*T discriminated 20 species. The PTP analysis indicated that *rpo*B‐*trn*C (36), *psa*A‐*ycf*3 (36), and *ycf*4‐*cem*A (35) had the highest estimated species numbers (Table 4). The GMYC analysis showed similar species numbers as the ABGD analysis with low threshold values, and *rpo*B‐*trn*C (27), *psa*A‐*ycf*3 (45), and *ycf*4‐*cem*A (29) had the highest estimated species numbers.

**Figure 3:**
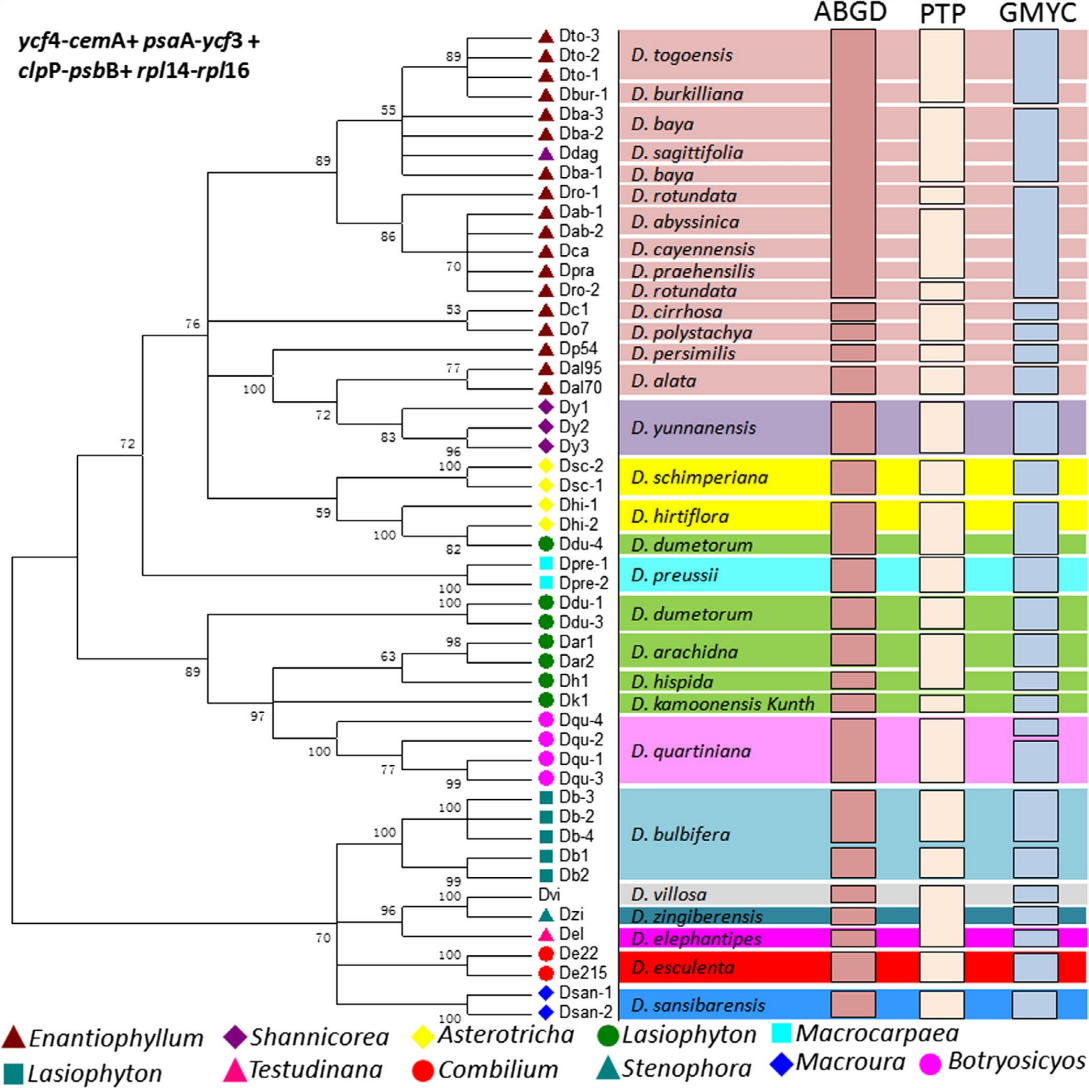
The phylogenetic tree constructed using maximum likelihood for *Dioscorea* species based on *ycf*4‐*cem*A + *psa*A‐*ycf*3 + *clp*P‐*psb*B + *rpl*14‐*rpl*16 (on the left) and summary of putative species delimitation drawn by BLAST, ABGD, PTP, and GMYC (on the right, one column per method)

Since *ycf*4‐*cem*A, *psa*A‐*ycf*3, *clp*P‐*psb*B, and *rpl*14‐*rpl*16 had sequences for 28 *Dioscorea* species, the combined sequences were used for phylogenetic tree construction via maximum‐likelihood analysis (Figure 3). The phylogenetic analysis showed that most individuals belonging to the same species tended to cluster together (node value >0.8), such as *D. bulbifera*, *D. arachidna*, *D. esculenta*, and *D. sansibarensis*. Moreover, species belonging to the same sections tended to group together, and the three sections—*Enantiophyllum*, *Shannicorea*, *Asterotricha*—have closer evolutionary relationships than the other sections. In the ABGD analysis, the pairwise genetic distance distribution showed two modes with a barcoding gap located between 0.1% and 1% (Table 4). The higher threshold levels suggested two species (1%), while low threshold values (0.2%) identified 23 species (Figure 3). The PTP analysis suggested 21 species with support value higher than 0.5. The GMYC analysis has predicted 24 effective candidate species, according to the lineage‐through‐time plot and the likelihood function estimated by the software R (*L*_0_ = 390.15, *L*_multiple_ = 399.27, *L*_ratio_ = 18.23, *p*‐value = .00011). Majority PTP and GMYC analysis shared similar species delimitation, except for species belonging to the *Lasiophyton* and *Enantiophyllum* sections.

## 4 DISCUSSION

An ideal DNA barcode should have high PCR amplification efficiency and cover regions with enough variability for species identification. Sun et al. (2012) applied *rbc*L, *mat*K, and *psb*A‐*trn*H to identify species within the *Dioscorea* genus and found *mat*K was the best DNA barcoding candidate, with a species discrimination rate of 23.26%. In this study, we made a thorough comparison of genic and intergenic regions of three *Dioscorea* chloroplast genome sequences to identify highly variable regions for DNA barcode development. We used 74 *Dioscorea* individuals from China and 47 *Dioscorea* SRAs from the NCBI database to estimate primer universality and species discrimination efficiency. A total of 29 species belonging to 11 sections were included in the analysis, among which Enantiophyllum Uline (14), Sect. Opsophyton Mine (2), Lasiophyton Uline (4), and Asterotricha (2) have more than one species. We selected seven pairs of primers (7/52) for further analysis which had high PCR amplification rates and distinct sequence variations between species. The intraspecific and interspecific variation analysis, along with different methods of species discrimination, indicated that these loci have divergent species discrimination efficiency.

DNA barcodes show a relatively variable species discrimination efficiency in different plants (Gogoi & Bhau, 2018; Hollingsworth et al., 2011; Liu et al., 2017; Sun et al., 2015, 2012), and more DNA barcodes for species‐level resolution have been developed and tested (Dong et al., 2015; Song et al., 2017). At present, the development of universal primers for highly variable regions relies on the availability of sequences for different species. New primers of ITS regions of plants with improved universality and specificity were designed based on 1,264,929 sequences of 18S, 5.8S, and 26S from the plant and fungus kingdoms (Cheng et al., 2016). The comparison of chloroplast genomes for genic and intergenic region between three *Dioscorea* species indicated that intergenic regions had more variable loci than genic regions and that conserved genic regions were suitable for primer design (Table 1). However, the primer sequences conserved between three *Dioscorea* species still have low ratios of universal amplification success across different species (Table S2). The low available numbers of *Dioscorea* chloroplast genomes sequences may limit the efficiency of primer design. With the growing of available chloroplast genome sequences, more efficient primers could be designed in silico.

Through analysis of a set of PCR amplicons from 73 *Dioscorea* individuals and 47 DNA SRA datasets of *Dioscorea* species, the seven selected loci showed significant variation for their interspecies distances. The *rpo*B‐*trn*C locus has the greatest average interspecies distances, and the Wilcoxon signed‐rank test indicated the same result (Tables 4 and 5). The *Dioscorea* genus contains more than 600 species, while *Dioscorea spp*. is used for unnamed wild *Dioscorea* species. Abundant efforts have been made to reveal the diversity and evolutionary relationship between *Dioscorea* species (Chaïr et al., 2005; Girma, Spillane, & Gedil, 2016; Hsu, Tsai, Chen, Ku, & Liu, 2013; Magwetindo, Zapfack, & Sonke, 2016; Mukherjee & Bhat, 2013; Ngwe, Omokolo, & Joly, 2015). Eleven *Dioscorea* sections were included in analysis in this study (Figure 3 and Tables S1 and S3). Phylogenetic analysis based on *ycf*4‐*cem*A, *psa*A‐*ycf*3, *clp*P‐*psb*B, and *rpl*14‐*rpl*16 loci produced clear clustering of most species to the sections, but species discrimination for species belonging to *Lasiophyton* and *Enantiophyllum* sections was not very accurate (Figure 3). This may be caused by the close evolutionary relationships between *Dioscorea* species in these sections.

With the growing availability of sequence information, species discrimination through molecular evidence is becoming both feasible and reliable. Plastid markers, such as *rbc*L, *mat*K, and *trn*H‐*psb*A, have been widely used with high amplification success in these regions (Hollingsworth et al., 2011). The internal transcribed spacers from nuclear ribosomal DNA, complete plastid genomes, and single copy nuclear genes have also been used in species discrimination (Cheng et al., 2016; Duarte et al., 2010; Song et al., 2017). In this study, we selected primers covering highly variable regions in the *Dioscorea* chloroplast genome. Although only seven pairs of primers had good amplification success, the success rates for species discrimination using these primers were high. Along with other research, in which primers for DNA barcodes have been designed based on available sequences, our results suggest that the growing amount of sequence information will greatly enhance the development of suitable DNA barcodes for taxonomy analysis and species delimitation.

## CONFLICT OF INTEREST

None declared.

## AUTHOR CONTRIBUTIONS

W.X., J.W., and D.H. conceived the presented idea. B.Z., W.X., and J.S. performed the data analysis and did the experiments. Y.D., W.W., W.T., and J.Z. collected the samples and did the DNA extraction. X.H., Y.X., and J.X. revised the manuscript. B.Z. and W.X. wrote the manuscript.

## Data Availability

Three chloroplast genome sequences for *Dioscorea* species are available in NCBI with accession numbers as KJ490011.1, EF380353.1 and KP899622.1. The sequences for *atp*F, *rpo*B‐*trn*C, *trn*D‐*trn*T, *psa*A‐*ycf*3, *ycf*4‐*cem*A, *clp*P‐*psb*B and *rpl*14‐*rpl*16 are listed in Table S1 and deposited in ENA (Accession: PRJEB29179, ERZ773993). The 47 SRAs for *Dioscorea* species are available in NCBI and accession numbers are listed in Table S3.
