# Supplementary figures and images for: Development of high‐resolution DNA barcodes for Dioscorea species discrimination and phylogenetic analysis
Source: Ecol Evol. 2019 Aug 22;9(18):10843–53. doi: 10.1002/ece3.5605 (PMC6787845; doi:10.1002/ece3.5605)

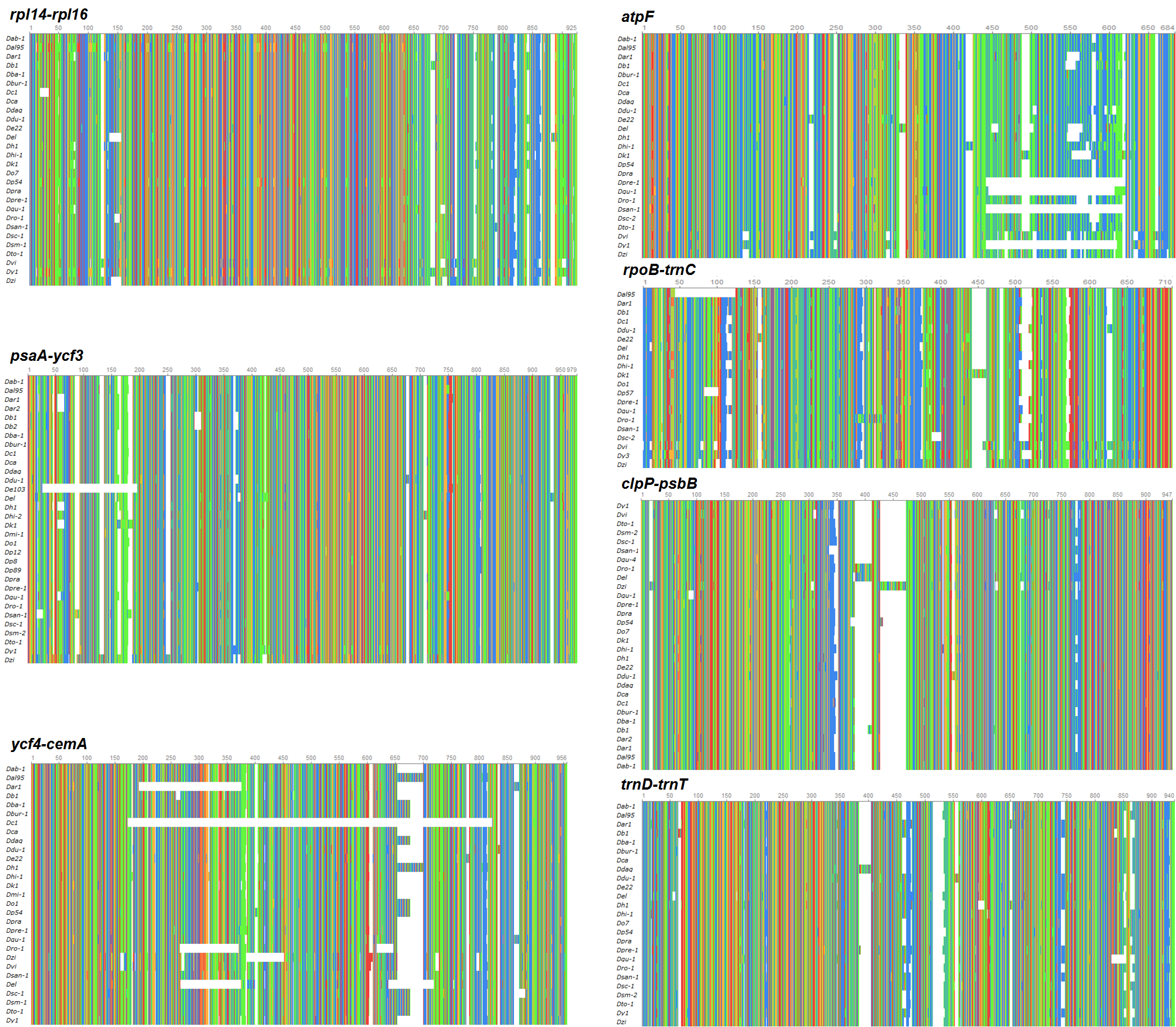

Supplement: Supplementary file 1 [file ECE3-9-10843-s001.tif]
