# Supplementary material for: Development of high‐resolution DNA barcodes for Dioscorea species discrimination and phylogenetic analysis
Source: Ecol Evol. 2019 Aug 22;9(18):10843–53. doi: 10.1002/ece3.5605 (PMC6787845; doi:10.1002/ece3.5605)
Supplement: Supplementary file 6 [file ECE3-9-10843-s006.docx]

**Supporting Information**

**Table S1** Information for *Dioscorea* species used for DNA extraction and sequencing in this study. Dashes represent PCR products were not sequenced.

**Table S2** Information for primers used in this study. Ten individuals from ten *Dioscorea* species (*D. alata*, *D. polystachya*, *D. esculenta*, *D. persimilis*, *D. bulbifera*, *D. cirrhosa*, *D. hispida*, *D. arachidna*, *D. kamoonensis Kunth*, and *D. yunnanensis*) were used to estimate the amplification efficiency.

**Table S3** Information for 47 SRAs of 18 *Dioscorea* species used for plastid related sequence assembly.

**Table S4** Sequence characters for the three chloroplast genome sequences from *Dioscorea* species used in this study.

**Figure S1** Sequence alignments of *atp*F, four intergenic sequences - *rpo*B*-trn*C, *ycf*4*-cem*A, *clp*P*-psb*B and *rpl*14*-rpl*16 and two containing both genic and intergenic sequences - *trn*D*-trn*T and *psa*A*-ycf*3 from analyzed samples of *Dioscorea* species. One individual for each *Dioscorea* species was selected and the sample names were listed as *D. zingiberensis* (Dzi), *D. elephantipes* (Del), *D. rotundata* (start with Dro), *D. cirrhosa* (Dc1), *D. bulbifera* (start with Db), *D. hispida* (Dh1), *D. arachidna* (Dar), *D. yunnanensis* (Dy1), *D. kamoonensis Kunth* (Dk), *D. persimilis* (start with Dp), *D. esculenta* (start with De), *D. alata* (start with Dal), *D. polystachya* (start with Do), *D. cirrhosa* (Dc1), D. abyssinica (start with Dab), *D. baya* (start with Dba), *D. burkilliana* (start with Dbur), *D. cayennensis* (start with Dca), *D. dumetorum* (start with Ddu), *D. hirtiflora* (start with Dhi), *D. minutiflora* (start with Dmi), *D. praehensilis* (Dpra), *D. preussii* (start with Dpre), *D. quartiniana* (start with Dqu), *D. sagittifolia* (Ddag), *D. sansibarensis* (start with Dsan), *D. schimperiana* (start with Dsc), *D. smilacifolia* (start with Dsm), *D. togoensis* (start with Dto), and *D. villosa* (Dvi). The numbers obove the sequences represent the position of the sequences. The flanking low quality parts of the amplicons were trimed and then aligned.
